# Variations in osteoporosis medication utilization. A population-based ecological cross-sectional study in the region of Valencia, Spain

**DOI:** 10.1371/journal.pone.0199086

**Published:** 2018-06-21

**Authors:** Gabriel Sanfélix-Gimeno, María-Lirios Juliá-Sanchis, Julián Librero, Salvador Peiró, Aníbal García-Sempere

**Affiliations:** 1 Center for Public Health Research (CSISP-FISABIO), Valencia, Spain; 2 Red de Investigación en Servicios de Salud en Enfermedades Crónicas (REDISSEC), Valencia, Spain; 3 Navarrabiomed, Navarra Biomedical Centre, Pamplona, Navarra, Spain; UiT The Arctic University of Norway, NORWAY

## Abstract

Little is known about the contextual variability in osteoporosis medication utilization. Our aims were 1) to describe variations in utilization and spending on osteoporotic medication between the Primary Care Health Zones (PHZ) of the Valencia region, Spain, 2) to analyze observed variations using Small Area Variation Analysis methods, and 3) to quantify the influence of the specialized care level on variations in utilization. We conducted a population-based cross-sectional ecological study of expenditure and utilization of five therapeutic groups marketed as osteoporosis treatments in Spain in 2009. The unit of analysis was the PHZ (in total 240) nested in the 23 Hospital Healthcare Departments (HHD) of the region of Valencia, covering a population of about 4.9 million inhabitants. Drug utilization was measured by dispensed Defined Daily Dose per 1000 women aged 50 years old and over and day (DID) per PHZ and cost was measured by the annual osteoporosis drug cost per woman aged 50 and older as well as the average price of DDD (Defined Daily Dose) in each PHZ. We calculated Indirect Standardized Drug Utilization Ratios (ISR) and we used Spearman’s correlation to analyze associations between the ISRs of the different therapies. The average osteoporosis drug consumption was 119.1 DID, ranging from 77.6 to 171.3 DID (2.2 times higher) between PHZs in the 5th and 95th percentiles. Annual expenditure also showed a two-fold variation among PHZs. Average prices of the DDD by therapeutic group showed very low or no variation, although they differed substantially among therapeutic groups. Regarding the standardized consumption of osteoporotic drugs, HHDs explained a substantial part (39%) of the variance among PHZs. In conclusion, there is considerable variability in the volume and choice of anti-osteoporotic treatments between PHZs. with HHDs explaining an important proportion of the variation in utilization. Interventions aimed at reducing variation to improve appropriate care should take into account both the PHZ and HHD levels of care.

## Introduction

Osteoporotic fractures are a major health problem in developed countries and have a corresponding impact on mortality, the quality of life and healthcare costs. Annually, 3.1 million osteoporotic fractures occur in Europe, equivalent to a loss of about two million disability-adjusted life years [1]. In Spain, it has been estimated that 714,000 osteoporotic fractures will occur in the next 10 years, mostly in women over 70 years old [2]. Targeting high-risk groups is a central component of cost-effective policies aiming at reducing the burden of osteoporotic fractures. Although different clinical approaches are currently available to lower the risk of fracture (e.g. lifestyle changes, the risk management of falls and several pharmacological treatments), there is considerable variability among clinical practice guidelines with regard to how to quantify that risk, who should receive densitometry testing, who should be treated, what treatment should be used and for how long [3–5].

The lack of strong, coherent, and widely accepted guidance for pharmacological treatment may result in a wide variation in the patterns of drug use among neighbouring populations with very similar demographic and epidemiological characteristics. While a significant amount of literature on Small Area Variations Analysis (SAVA) in surgical and medical hospitalizations and healthcare spending has been an essential element to interpret the behaviour of healthcare providers and to define public policies [6–9], studies examining geographic variations in pharmaceutical utilization have been scarce [10,11] until recent years [12–15]. Besides, very few have addressed–and then only partially–variability in osteoporosis medication utilization [16,17].

Beyond the evidence provided by clinical trials, the recommendations of clinical practice guidelines and the agreements reached by expert consensus panels, treatment decisions and the choice of a particular drug can be influenced by patient characteristics, physician and organizational factors, pharmaceutical promotion and healthcare system characteristics [18]. For instance, Spain is a country with one of the lowest incidence of osteoporotic fractures in the world, but utilization rates of osteoporosis medication are among the highest. In addition, considering that the treatment of osteoporosis is managed by primary care physicians and several other medical specialists (rheumatologists, gynaecologists and orthopaedic surgeons among others), to know to what extent the different levels of care explain geographical variations seems of special interest.

The aims of this study are: 1) to describe variations in utilization and expenditure on osteoporosis medication between the Primary Care Health Zones (PHZ) of the Valencia region, Spain, 2) to analyze observed variations using SAVA methods, and 3) to quantify the influence of the specialized care level on variations in utilization.

## Materials and methods

### Design

Population-based cross-sectional ecological study of expenditure and utilization of five therapeutic groups marketed as osteoporosis treatments in Spain.

### Population

The units of analysis were the 240 PHZs nested in 23 Hospital Healthcare Departments (HHD) in the region of Valencia, Spain, in 2009. These PHZs, after excluding non-residents and people not entitled to pharmaceutical benefits, covered a population of about 4.9 million inhabitants in 2009. The population of study were women aged 50 and over (N = 898,544; 18%).

### Setting

The region of Valencia is located on the Mediterranean east coast of Spain and provides universal public healthcare coverage. [19] In 2009, healthcare was free of charge, except for drugs for non-retired people, who had a 40% copayment. Hospital and primary care is financed by the regional government budget and at that time was organized into 23 HHDs (geographically delimited territories between 75,000 and 500,000 inhabitants, most of them having between 150,000–250,000 inhabitants). HHDs are served by one public hospital that provides specialized inpatient and outpatient care to the PHZs within its demarcation, and PHZs (small geographical areas of between 1,000 and 65,000 inhabitants, most of them having between 10,000 and 25,000 inhabitants) are typically served by one Primary Healthcare Center (PHC) with a stable team of doctors, nurses and other healthcare professionals. Due to these organizational characteristics (geographical organisation and minimal accessibility barriers) patients receive most of their primary care from the PHC of the PHZ they belong to, and most of their specialized care, including outpatient visits, from the hospital and its specialized outpatient clinics in the corresponding HHD.

### Data sources

Utilization and expenditure data were supplied by the Valencia Health Agency (VHA) from its drug dispensation data files, containing information on the pharmacy claims reimbursed in 2009 aggregated at the PHZ level (only aggregated claims data were available for research for the whole region in 2009). This database includes information about the volume of dispensed drugs (detailing commercial brands, generic names, strengths, size of the packages and retail prices), patient’s coverage (full coverage or 40% copayment), and identifies the PHC, the PHZ and the HHD providing the service. The population in each PHZ and HHD was obtained from the VHA register, the so-called Population Information System.

### Therapeutic groups

We selected all drugs marketed in Spain in 2009 for the treatment of osteoporosis categorized into five Anatomical Therapeutic Chemical (ATC) Classification groups: M05BA and M05BB (biphosphonate and combinations, including alendronate, risendronate, ibandronate, etidronate and alendronate/vitamin D), M05BX (strontium ranelate), G03XC (raloxifene), H05AA (parathyroid hormone and teriparatide) and H05BA (synthetic salmon calcitonin and elcatonin). Data on zoledronic acid (only administered within the hospital in Spain) was not available because of the lack of in-hospital medication claims data.

### Main measure of drug utilization

Defined Daily Dose (DDD) per 1000 inhabitants and day (DDD/1000 inhabitants/Day, or DID) dispensed in each PHZ based on the pharmaceutical claims reimbursed by the Valencia Health Department. The DDD is a technical unit of measurement used in drug utilization studies and assigned by the WHO Collaborating Centre for Drug Statistics Methodology [20], equivalent to the average daily maintenance dose of a drug in its main indication for adults. The DDD/1000 inhabitants/Day or DID *[(total consumption in a population aggregated in DDD x 1000) / (365 days x number inhabitants in the population)]* is a rough estimate of the number of people per 1000 inhabitants who at any given moment are consuming a specific drug. Because of the strong sex-age related use of osteoporosis medication (according to non-published data from the Department of Health, men only account for 5% of treatments, and for women only 5% goes to those under 50 years old) we assigned all consumption to the population of women aged 50 years old and over.

### Other measures

1) The pharmaceutical expenditure on osteoporosis medication at retail price per woman aged 50 and over and year; 2) The average price of the DDD calculated as pharmaceutical expenditure divided by dispensed DDD; 3) the age-copayment *Indirect Standardized Drug Utilization Ratio* (ISR), obtained as the ratio of the observed DDD dispensed in each PHZ to an estimate of the expected DDD in the same PHZ, given age and copayment specific drug utilization rates from a reference population.

### Ethical aspects

The study, observational in design, using retrospective ecological non-individual non-identifiable data, did not require Ethics Committee approval.

### Analysis

First, we estimated the DID by PHZ. Second, expenditure in each therapeutic class was aggregated by PHZ and divided by the number of women aged 50 and older and by DDD dispensed to obtain the annual anti-osteoporotic medication cost per woman aged 50 and older and the average price of DDD in each PHZ. Third, *Indirect Standardized Drug Utilization Ratios* in each PHZ, similarly to Standardized Mortality Ratios (SMR) [21,22], were estimated for each therapeutic class as ISR = observed/expected = Σ_i_ DDD_i_/ Σ_i_ DUR^R^_i_ *N*_*i*_ where DUR^R^_i_ is the drug utilization rate in the *i*th age-copayment group in a reference population in which these parameters were known. We used the available specific age-copayment rates from the Madrid region but recalibrated to the observed total consumption in the Valencia region (for more details, see S1 Appendix). The indirect method used does not allow the comparing of one PHZ with another, but does permit a comparison with the reference pattern that by construction is equivalent to the average utilization rate of the region of Valencia for the respective drug. Therefore, an ISR such as 1.50 for a given PHZ is interpreted (like for relative risks) as meaning that this PHZ has a consumption of the respective drug 50% higher than the Valencia region average. While an ISR such as 0.67 corresponds to a decrease of 50% in consumption with respect to the Valencia region average.

Fourth, we carried out a descriptive analysis of osteoporosis medication utilization (crude and standardized), pharmaceutical expenditure per woman and price by DDD, presenting estimated values for the mean, median, and the values of the PHZs in percentiles (P) 5, 25, 75 and 95.Variability among PHZs was analyzed using SAVA statistics [23] but, in order to reduce the influence of a few areas with extreme values, we excluded (except otherwise noted) the PHZs outside the 5^th^ and 95^th^ percentile of the corresponding distribution. The statistics used include the high-low ratio (the ratio between the PHZs in P5 and P95, also known as the extremal quotient; EQ5-95), the interquartile ratio (the ratio between PHZs in P25 and P75; IQR25-75), the coefficient of variation (CoV5-95) and the weighted coefficient of variation (CoVW5-95), similar to CoV5-95 but weighted for the size of the population in each area (see S1 Appendix). The ISRs in each PHZ and therapeutic class were also displayed as geographical maps.

Finally, Spearman’s correlation was used to analyze associations between the ISRs of the different therapies. A one-way ANOVA random-effects analysis using the HHD as a factor was used to estimate the proportion of between-PHZ variance explained by HHD level, which provides several statistics of within groups homogeneity (Intraclass correlation coefficient, the estimated reliability of the group mean score, both with similar interpretation, the greater the value the greater the homogeneity within HHD). Additionally, a multiple linear regression analysis was used to assess the variance explained by the ISRs of each therapeutic group on the total pharmaceutical expenditure on osteoporosis medication. All the analyses were performed using the STATA 11.0 (Stata Corp, College Station, Texas) statistical software and R (Free Software Foundation’s GNU General Public License, Boston).

## Results

The average consumption of osteoporosis medication in the Valencia region in 2009 was 119.1 DID, ranging from 77.6 to 171.3 DID (a 2.2-fold difference) between PHZs in the 5th and 95th percentiles (Table 1). Biphosphonates represented 70.3% of the overall consumption (88.4 DID) followed by raloxifene (14.6%; 16.8 DID), strontium ranelate (8.6%; 10.4 DID), calcitonins (4.5%; 5.7 DID) and parathyroid hormones including teriparatide (2.0%; 2.4 DID). Regarding variability by therapeutic groups, biphosphonates showed the lowest variation (a 2.5 times higher consumption in the PHZ in the 95th percentile compared to that in the 5th percentile), with calcitonins and parathyroid hormones being the highest (around a 9-fold higher consumption between PHZs at the 95th and 5th percentiles). Age and copayment standardization (Table 1) did not substantially modify the variability among PHZs. Maps of ISR per PHZ show the geographical distribution of variation. In many cases the Primary Care Zones nested in the same Health Department adopt similar figures (S2 Appendix). Therapeutic group–specific Spearman correlations in standardized consumption (S3 Appendix) were significant and positive but weak (range: 0.14–0.35), except for two non-significant pairs (raloxifene/biphosphonates and strontium ranelate/parathyroid hormones).

**Table 1:** Use of osteoporosis medication among women ≥ 50 years by Primary Healthcare Zones in the region of Valencia, 2009.

	Biphosph.	Strontium R	Raloxifene	Parathyr. H	Calciton.	All
***Defined Daily Dose/1000 inhabitants/day***						
Mean	83.76	10.22	17.39	2.33	5.40	119.10
SD	20.43	4.24	5.96	1.36	3.14	25.65
P5	52.58	3.53	8.10	0.53	1.38	77.61
P25	73.93	6.97	12.02	1.29	3.34	105.60
Median	86.52	10.05	15.86	2.06	4.81	121.39
P75	99.49	13.25	20.20	3.19	6.91	139.19
P95	130.92	18.44	29.10	5.38	12.57	171.34
EQ5-95	2.49	5.23	3.59	10.13	9.08	2.21
IQR25-75	1.35	1.90	1.68	2.47	2.06	1.32
CV5-95	0.20	0.37	0.30	0.52	0.48	0.17
CVW5-95	0.20	0.37	0.29	0.53	0.50	0.17
***Indirect Standardized Drug Utilization Ratios (reference pattern = 1)*.**						
P5	0.61	0.33	0.48	0.21	0.27	0.63
P25	0.85	0.68	0.69	0.54	0.61	0.87
Median	1.02	0.95	0.91	0.88	0.88	1.02
P75	1.17	1.28	1.17	1.35	1.28	1.16
P95	1.49	1.81	1.80	2.22	2.25	1.45
EQ5-95	2.43	5.46	3.75	10.68	8.41	2.29
IQR25-75	1.37	1.87	1.69	2.50	2.11	1.34
CV5-95	0.20	0.37	0.30	0.51	0.47	0.17
CVW5-95	0.21	0.38	0.28	0.52	0.49	0.18

SD: Standard Deviation; P: Percentile; EQ: Extremal Quotient; IQR: Interquartile Ratio; CV: Coefficient of Variation; CVW: Weighted coefficient of variation.

The annual expenditure on osteoporosis medication (Table 2) was €59.7 million, equivalent to an expenditure of €66.4 per woman aged 50 and over. Expenditure varied 2.2-fold between PHZs in the 5th and 95th percentiles, ranging from €43.8 to €98.5. Biphosphonates, despite accounting for 70.3% of the overall consumption, only reached 53.1% of the overall expenditure on osteoporosis medication, while parathyroid hormones, representing only 2.3% of the overall consumption accounted for 18.3% of osteoporosis pharmaceutical expenditure. Biphosphonates showed the lowest variation in expenditure (2.5-fold between PHZs at the 95th and 5th percentiles), followed by raloxifene (EQ5-95: 3.6) and strontium ranelate (EQ5-95: 5.3), while calcitonines (EQ5-95: 8.6) and parathyroid hormones (EQ5-95: 9.9) showed the highest variation. Average prices of the DDD by therapeutic group (Table 3) showed very low or no variation, although they differed substantially between therapeutic groups, ranging from 1.1€/DDD for bisphosphonates to 14.3€/DDD for parathyroid hormones. The overall mean average price per DDD in osteoporosis medication was €1.5, showing low variation among PHZs (EQ5-95: 1.4).

**Table 2:** Osteoporosis medication expenditure in euros per woman aged 50 and over by Primary Healthcare Zones in the region of Valencia, 2009.

	Biphosph.	Strontium R	Raloxifene	Parathyr. H	Calciton.	All
***Drug expenditure per woman aged 50 and over***						
**Mean**	34.60	6.58	7.80	12.19	5.29	66.44
**SD**	8.53	2.73	2.67	7.10	2.96	16.03
**P5**	21.43	2.27	3.64	2.86	1.41	43.80
**P25**	30.26	4.49	5.39	6.82	3.39	56.85
**Median**	35.75	6.47	7.11	10.82	4.73	66.88
**P75**	41.49	8.53	9.06	16.65	6.65	76.99
**P95**	53.04	11.87	13.05	28.30	12.14	98.46
**EQ5-95**	2.47	5.23	3.59	9.88	8.64	2.25
**IQR25-75**	1.37	1.90	1.68	2.44	1.96	1.35
**CV5-95**	0.20	0.37	0.30	0.52	0.47	0.19
**CVW5-95**	0.21	0.37	0.28	0.53	0.48	0.20
***Average Price per Defined Daily Dose (euros)***						
**Mean**	1.13	1.76	1.23	14.29	2.70	1.53
**SD**	0.04	0.00	0.00	0.00	0.08	0.14
**P5**	1.07	1.76	1.23	14.29	2.53	1.30
**P25**	1.11	1.76	1.23	14.29	2.64	1.40
**Median**	1.13	1.76	1.23	14.29	2.70	1.50
**P75**	1.15	1.76	1.23	14.29	2.76	1.62
**P95**	1.20	1.76	1.23	14.29	2.85	1.82
**EQ5-95**	1.13	1.00	1.00	1.00	1.12	1.40
**IQR25-75**	1.04	1.00	1.00	1.00	1.04	1.16
**CV5-95**	0.03	0.00	0.00	0.00	0.03	0.08
**CVW5-95**	0.02	0.00	0.00	0.00	0.02	0.08

SD: Standard Deviation; P: Percentile; EQ: Extremal Quotient; IQR: Interquartile Ratio; CV: Coefficient of Variation; CVW: Weighted coefficient of variation.

**Table 3:** Variance at the PHZ level explained by the Hospital Health Department in the region of Valencia, 2009.

	Biphosph.	Strontium R	Raloxifene	Parathyr. H	Calciton.	All
***Indirect Standardized Drug Utilization Ratios***						
ICC	0.33	0.35	0.33	0.58	0.37	0.39
95%CI ICC	0.16–0.50	0.18–0.52	0.16–0.51	0.41–0.75	0.19–0.54	0.21–0.57
SD between HHD	0.16	0.27	0.24	0.47	0.40	0.15
SD within HHD	0.23	0.36	0.33	0.40	0.52	0.19
Reliability	0.84	0.85	0.84	0.94	0.86	0.87
***Expenditure per woman 50 years old and over***						
ICC	0.34	0.38	0.33	0.57	0.37	0.54
95%CI ICC	0.17–0.51	0.20–0.55	0.16–0.50	0.40–0.74	0.20–0.55	0.37–0.62
SD between HHD	5.66	1.82	1.76	5.97	2.13	12.93
SD within HHD	7.95	2.35	2.48	5.18	2.76	11.82
Reliability	0.84	0.86	0.84	0.93	0.86	0.93

Random effects ANOVA one-way analysis P<0.0001 for all analysis. VPC: ICC: Intraclass correlation coefficient; CI: confidence interval; SD: Standard Deviation (in ISR scale or euros scale), HHD: Hospital Health Department.

Regarding the standardized consumption in osteoporosis medication, HHDs explained a substantial part (39%) of the variance between PHZs (Table 3), ranging from 33% (biphosphonates or raloxifene) to 58% (parathyroid hormones) depending on the therapeutic group. However, the standard deviations between HHDs were lower than those within HHDs, except for parathyroid hormones where the within HHD standard deviation was higher than the between HHD deviation (0.47 and 0.40, respectively). Also, the high reliability values reflect a high intragroup correlation between PHZs from the same HHD. S4 Appendix shows the distribution of variation per HHD.

Regarding expenditure, the random-effects analysis of variance showed a similar pattern of results except for the overall pharmaceutical expenditure, where 54% of the variance between PHZs was explained by the HHD level. The standard deviation between (€12.9) and within (€11.8) HHD was nearly equivalent. The therapeutic group–specific standardized consumption explained 95% of the total expenditure on osteoporosis medication, with biphosphonates and parathyroid hormones being the most influential drugs (Table 4).

**Table 4:** Relation between ISR and total expenditure on osteoporosis medication. Multivariate Regression Analysis.

	Coef.	Standard ß	p
ISR Biphosphonates	34.51	0.55	<0.001
ISR Strontium R.	5.99	0.15	<0.001
ISR Raloxifene	6.92	0.16	<0.001
ISR Parathyroid H.	12.94	0.46	<0.001
ISR Calcitonines	5.06	0.19	<0.001
Constant	1.85		0.095

n = 240; p<0.0001; Adjusted R^2^ = 0.954; ISR: Indirect Standardized Drug Utilization Ratios

## Discussion

Our study has several findings of interest. First of all, it shows considerable variability between PHZs, with ratios in the 95th percentile more than doubling those in the 5th percentile for osteoporosis medications altogether, and reaching up to a 10-fold difference for some costly drugs such as parathyroid hormones. These figures are higher than those reported in previous studies in Portugal (2004) [16] and Norway (2007) [17]. While Spain is one of the European and worldwide countries with a lower incidence of osteoporotic fractures [24,25], osteoporosis medications are among the most widely prescribed drugs (a recent report analyzing variability in the consumption of several drugs in 15 developed countries, including the U.S., Canada, and several European countries, placed Spain as the country with the highest utilization of osteoporosis medication [26]) and with temporal trends showing a very rapid growth in their utilization [27]. It is possible that greater utilization also implies greater variability.

Second, the magnitude of the differences in osteoporosis medication utilization between territories is barely modified by standardizing for age and copayment, suggesting a limited role for morbidity (age is a good proxy for the prevalence of osteoporosis) or cost-sharing as a source of variations between geographical territories.

Third, variation in drug utilization is transferred almost entirely to variation in expenditure per capita (in this case, per woman over 50) and furthermore the rates of utilization explained almost all the variance in drug spending. The different amounts of medications prescribed in each territory, rather than their price, are the main source of variation in osteoporosis medication expenditure. The explanation for this divergence should be sought in the homogeneity of prices in Spain of the most widely used osteoporosis medications (derived from the system of price regulation including the reference pricing system, and the existence of several drugs still under patent at the time of the study), which shifts the weight of the variability in expenditure to differences in utilization rates. This may not occur in other drug groups with a greater variety in prices (in countries with different systems of price regulation) or with increased consumption in high-cost drug groups. Finally, the public healthcare provision has an inflexible geographical organisation system in two levels of care with primary care health zones nested in hospital/specialized areas of service with very little across border flow. This becomes a particularly appropriate environment for analyzing the relationship between these two levels of care in drug pattern variations, and it is one of the aspects of interest in our study. The HHD level accounted for considerable share of the variation, with variations between HHDs being higher than variations within HHDs for all drugs except for parathyroid hormones. This is consistent with the fact that the prescription of this therapeutic class is mainly led by specialists (HHD level), in contrast to other classes such as biphosphonates, which is a drug class widely prescribed in both levels of care. A study conducted in the U.S. using Medicare drug and nondrug claims and bigger territorial demarcations (306 Hospital Referral regions [HRRs] and 3436 local hospital service areas [HSAs] for all the U.S.) found that 41% of the variation in drug spending between HSAs was explained by HRRs. A high proportion of low-spending HSAs were included in high-spending HRRs and vice-versa, limiting the potential of policy interventions at HRR level without affecting HSAs with differential patterns of behaviours regarding to the HRR in which they are included [15]. However, in the VHA where the managerial structures of primary and specialty care are integrated at the HHD level, these policies should have fewer limitations. In any case, the existence of PHZs with differential behaviour suggests that interventions aimed at reducing variations in care should consider the PHZ component, in order to avoid a potential restriction of health resources in populations that could suffer underuse problems, even if nested in a HHD with overuse problems. For instance, and on the basis of our findings with regard to the concerning variability in the use of parathyroid hormone and teriparatide, the Valencia Health Agency implemented in 2012 a system level intervention–both at HHD and PHZ levels—to rationalize the use of these therapies in the region [28].

Our findings are subject to several limitations. First, the ecological design limits both transfer of the results from the geographically aggregated population basis to the individual basis and the causal connections of the associations detected. Second, we used pharmacy claims data providing an accurate picture of dispensed drugs, which may be an imprecise measure of doctors’ prescriptions or of the medication truly taken by patients. Third, the Valencia region is a popular tourist destination and to some extent part of treatments–especially in coastal “sea and sand” PHZs–could have been filled by patients from other Spanish regions or indeed other countries. Fourth, indirect adjustment for age and copayment may be insufficient to control for differences in the risk of being treated with anti-osteoporotic drugs, and other variables not available in our study (e.g. people with malabsorption syndrome, treated with corticosteroids, with prior osteoporotic fracture, and other) could improve the adjustment. Fifth, we assigned all DDDs consumed to women aged 50 and over. Although this is a reasonable strategy because it is the population group that consumes the vast majority of osteoporosis medication variations between PCZs in utilization in men or young women could affect our estimates of variability for the group of women aged 50 or older, albeit in discreet manner. Last, the results presented correspond to 2009, and may not be generalizable to present-day Valencia (the use of anti-osteoporotic drugs in Spain may have been affected by several factors, from safety warnings about bisphosphonates to the expiration of some patents, warnings, restrictions of use and/or withdrawal of some drugs, the incorporation of new drugs, as well as policies developed in response to the economic crisis or the impoverishment of the population itself). A very recent study [29] shows trends in osteoporosis medication use until 2013, being the most striking a significant decrease in consumption during the economic crisis (just like it happened with drugs for other diseases). And, although the amounts consumed and its distribution among the different drugs may have changed, to our knowledge no other study to date has analysed the variability in osteoporosis medication utilization in the Spanish NHS nor in other settings, and thus, our study provides novel and contemporary information.

In recent decades a substantial body of evidence has highlighted the existence of wide geographical variation in health care utilization and spending that is not driven by patient characteristics and is not associated with the quality of care or patient outcomes [6–15]. This evidence has been interpreted both as a marker of inefficient resource use and as an opportunity for improving the quality and value of health care. Our study, focused on osteoporosis medication utilization and spending, shows results consistent with this previous literature. It has been suggested that clinical trials generated to obtain market authorizations produce a large body of evidence that, in turn, reduces uncertainty (and variability) in the use of drugs. But in many cases there is still significant unresolved uncertainty which results in variability in drug utilization in the real world. In the case of osteoporosis medication, previous studies suggest major disagreement among clinical practice guidelines and a massive use of anti-osteoporotic drugs in women under 65 years old with a low-risk of fracture, while there is significant underuse in women (and men) with a high-risk of fracture, including those who have already suffered a major osteoporotic fracture [30–33]. Large geographical variations in drug utilization rates could be useful to recognize inconsistencies in the clinical management of specific health problems and to guide policy proposals for targeting areas with problems of misuse. Our study suggests that these policies should be addressed to a system level component (including both the primary and the specialized level of care) and with specific strategies oriented to price and/or volume according to drugs and contexts.

## Supporting information

S1 Appendix Small area variation statistics and indirect standardization. (DOCX)

S2 Appendix Maps of indirect standardized drug utilization ratios of osteoporosis medication use among women ≥ 50 years by Primary Healthcare Zones in the region of valencia, 2009. (DOCX)

S3 Appendix Spearman correlations between Primary Healthcare Zones indirect standardized drug utilization ratios of the osteoporosis medication (Valencia region, 2009). (DOCX)

S4 Appendix Median odds ratio of osteoporosis medication use (DDD/1000/Day) among women ≥50 years by hospital departments in the Valencia region, 2009. (DOC)

S1 Dataset Data analysis. (DTA)

S2 Dataset Population age & copayment. (DTA)
