# Supplementary material for: Variations in osteoporosis medication utilization. A population-based ecological cross-sectional study in the region of Valencia, Spain
Source: PLoS One. 2018 Jun 21;13(6):e0199086. doi: 10.1371/journal.pone.0199086 (PMC6013112; doi:10.1371/journal.pone.0199086)
Supplement: S1 Appendix — (DOCX) [file pone.0199086.s001.docx]

| Sanfélix-Gimeno G, Juliá-Sanchís ML, Librero-López J, Peiró S, García-Sempere A.  **Variations in osteoporosis medication utilization. A population-based ecological cross-sectional study in the region of Valencia, Spain** |
| --- |
|  |
| **S1 Appendix** |
| **Small Area Variation Statistics and indirect standardization** |

**Small Area Variation Statistics**

Small Area Variation Analysis (SAVA) is a method used in health services research to describe how rates of healthcare utilization vary across geographic areas. Two groups of statistics of variation are commonly used: those that describe the distribution of rates and those that use differences between expected and observed cases based on indirect standardization. Statistics among the former usually include the high-low ratio or extremal quotient (EQ) and the unweighted (CV) and weighted (CVw) coefficients of variation (table A1.1).^[1]^

| Table A1.1. Formulation of the small area variation statistics | |
| --- | --- |
| Extremal quotient (EQ) |  |
| Coefficient of Variation (CV) |  |
| Weighted Coefficient of Variation (CVW) |  |
| *Crude rates (DDD/1000p/Day) for each i-th PHZ are denoted by DSRi for i=1,…, I.* | |

- The **Extremal Quotient** (EQ; also known as high-low ratio) is the ratio between the highest and the lowest of the observed rates for each *i-th* PHZ.
- The **Coefficient of Variation** (CV) is the quotient between the standard deviation between PHZ rates and the mean PHZ rate.
- The **Weighted Coefficient of Variation** (CVw) is the quotient between the standard deviation between PHZ rates and the average PHZ rate, weighted by the size (population) of each PHZ.
- The **Indirect Standardized Drug Utilization Ratios** (ISR) were estimated as the quotient of the observed to the expected number of cases (ISR_i_ = y_i_/e_i_ for the *i-th* PHZ). This quotient is the maximum-likelihood estimator of r_i_ (the unknown relative risk of drug prescription in the PHZ) under the assumption that y_i_ ~Poisson(e_i_r_i_) independently for each *i-th* PHZ.

In all small area variation analysis in this paper (unless otherwise indicated) we exclude 5% of the extreme values for each tail to avoid that few unusual values (which are normally associated with territories with a small population) have a disproportionate impact on the statistics, and because occasionally the lower tail can include Primary Healthcare Zone (PHZ) with 0 cases that makes it difficult to calculate the statistics of variability.

**References**

^[1]^ Ibáñez B, Librero J, Bernal-Delgado E, Peiró S, López-Valcarcel BG, Martínez N, et al. Is there much variation in variation? Revisiting statistics of small area variation in health services research. BMC Health Serv Res. 2009;9:60. Available in: https://www.ncbi.nlm.nih.gov/pmc/articles/ PMC2676262/

**Indirect Standardized Drug Utilization Ratios**

Due to the strong sex-age gradient present in the prevalence of osteoporosis and in the related use of anti-osteoporotic drugs, the adequate comparison of the consumption of these medicines between different territories requires adjusting for the demographic structure of each territory. In the same way, copayment is an important determinant of drug consumption and comparisons between territories also require adjustment for differences in the proportion of people subject to copayment.

As information on the specific consumption rates of anti-osteoporotic medication by sex, age group and copayment status was available neither in the different PHZ, nor for the whole region of Valencia, we used indirect standardization. Indirect standardization allows for the calculation of the expected rates for our index population (women aged 50 and over for every PHZ in the region of Valencia), given age, sex and copayment status specific rates from a reference population. Our reference population was the region of Madrid, where these rates were available. Accordingly, we used Madrid rates to construct a valid reference pattern to estimate the adjusted consumption in each PHZ in our study.

To that end, we applied the specific consumption rates for the female population in the region of Madrid, by age groups (five-years groups from 50 to 85 and over) and copayment status (yes/no to each of the age-copayment stratum of the population of Valencia, recalibrating them to meet the condition of generating an expected total consumption equivalent to the observed consumption for the whole region of Valencia. In this way we obtained the expected consumption per PHZ.

Indirect Standardized Drug Utilization Ratios (ISR) for each PHZ were obtained by relating the observed consumption (observed DDD) with the expected DDD, representing the foreseeable consumption in each PHZ under the assumption that populations behave similarly to the reference population in terms of relative consumption. Contrary to direct methods, indirect standardization does not allow for the comparison between PHZs, given that constant specific rates (those of the reference population) are applied on the demographic pyramid of each PHZ. However, ISR does allow for the comparison of each PHZ with the pattern of reference that, by construction, approximates the average of all the PHZs studied (showing the adjusted distribution of variation around the mean PHZ consumption).

The database “PONE-D-17-22089 POPULATION AGE&COPAYMENT.dta” (Stata database) includes the population stratum for each PHZ used for building the ISRs.
