# Supplementary material for: Variations in osteoporosis medication utilization. A population-based ecological cross-sectional study in the region of Valencia, Spain
Source: PLoS One. 2018 Jun 21;13(6):e0199086. doi: 10.1371/journal.pone.0199086 (PMC6013112; doi:10.1371/journal.pone.0199086)
Supplement: S2 Appendix — (DOCX) [file pone.0199086.s002.docx]

| Sanfélix-Gimeno G, Juliá-Sanchís ML, Librero-López J, Peiró S, García-Sempere A.  **Variations in osteoporosis medication utilization. A population-based ecological cross-sectional study in the region of Valencia, Spain** |
| --- |
|  |
| **S2 Appendix** |
| **Indirect Standardized Drug Utilization Ratios of osteoporosis medication use among women ≥ 50 years by Primary Healthcare Zones in the region of Valencia, 2009.** |
|  |
| Figure A2.1. Maps of Indirect Standardized Drug Utilization Ratios of Biphosphonates use among women ≥ 50 years by Primary Healthcare Zones in the region of Valencia, 2009. |
| 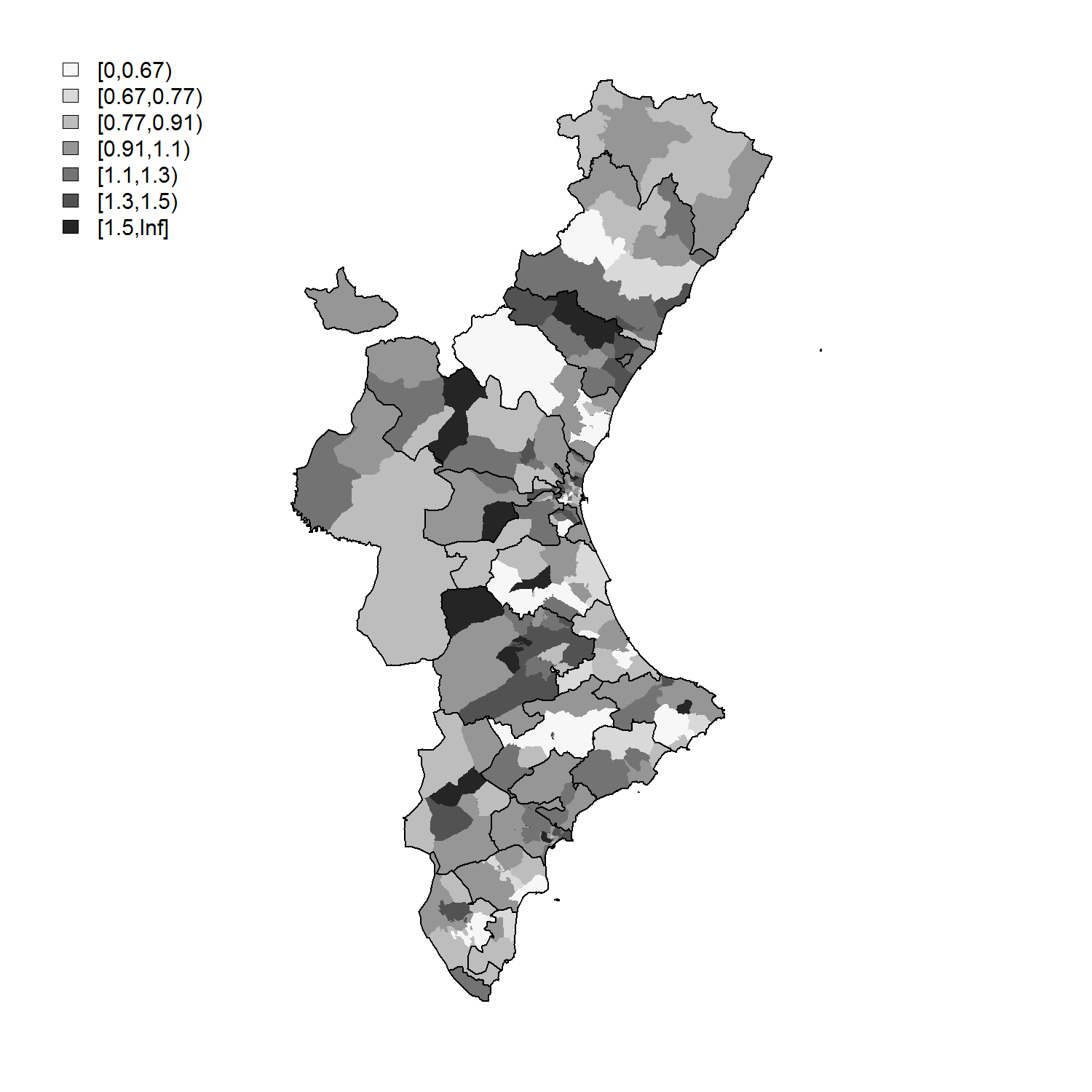 |
| *The lines on the map represent the geographical boundaries of the different Hospital Healthcare Departments (HHD). The cut-off points of Indirect Standardized Drug Utilization Ratios should be interpreted (like relative risks) on a ratios’ scale, corresponding to relative increases of consumption of 10% (1.10), 30% (1.30), 50% (1.50) and >50% (>1.50) with respect to the average of the Valencia region, and its symmetric negative -10% (0.91), -30% (0.77), -50% (0.67) and <-50 (<0.67). Map constructed upon raw maps from the Valencia Cartographic Institute (CC BY 4.0 © Institut Cartogràfic Valencià, Generalitat).* |

| Figure A2.2. Maps of Indirect Standardized Drug Utilization Ratios of Strontium Ranelate use among women ≥ 50 years by Primary Healthcare Zones in the region of Valencia, 2009. |
| --- |
| 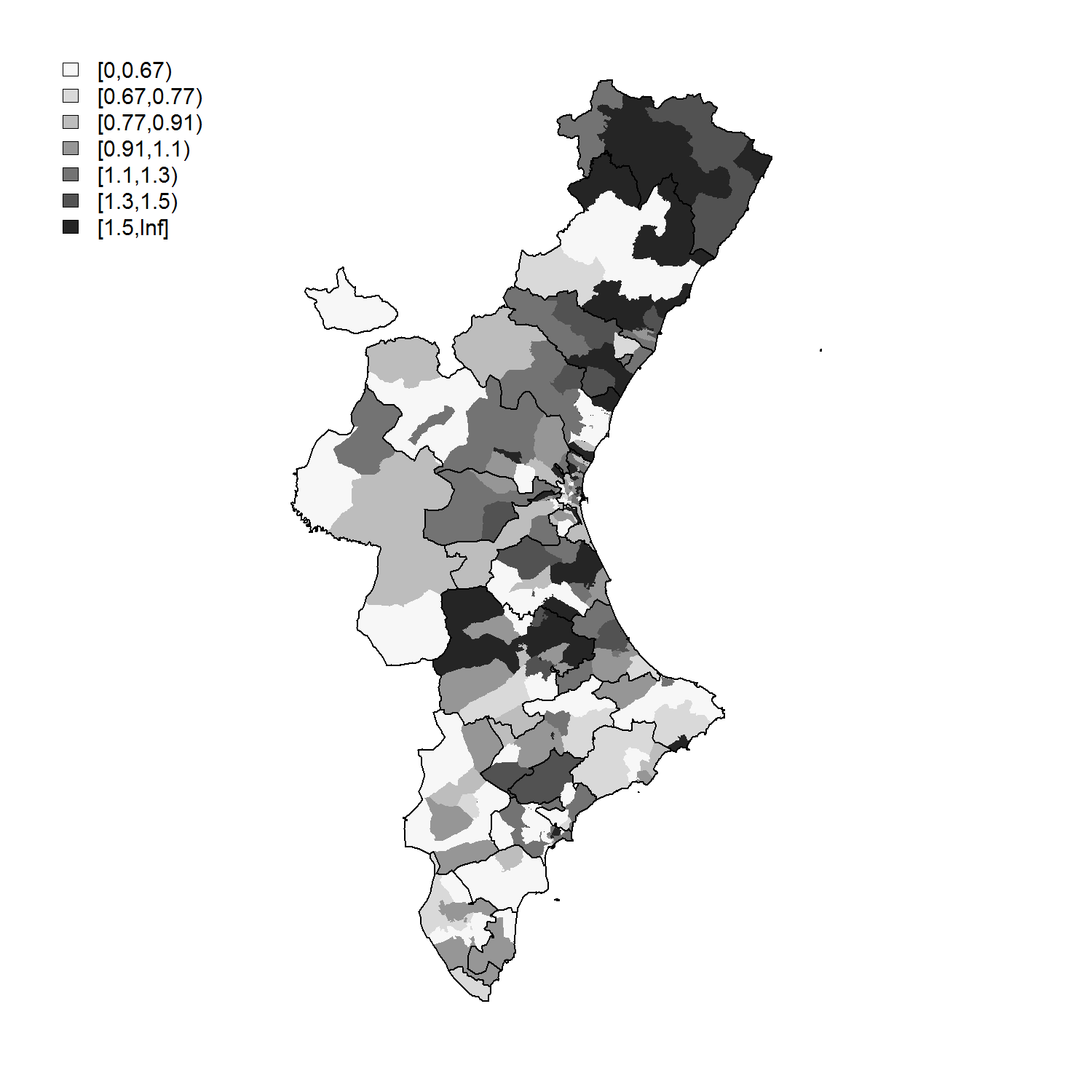 |
| *The lines on the map represent the geographical boundaries of the different Hospital Healthcare Departments (HHD). The cut-off points of Indirect Standardized Drug Utilization Ratios should be interpreted (like relative risks) on a ratios’ scale, corresponding to relative increases of consumption of 10% (1.10), 30% (1.30), 50% (1.50) and >50% (>1.50) with respect to the average of the Valencia region, and its symmetric negative -10% (0.91), -30% (0.77), -50% (0.67) and <-50 (<0.67). Map constructed upon raw maps from the Valencia Cartographic Institute (CC BY 4.0 © Institut Cartogràfic Valencià, Generalitat).* |

| Figure A2.3. Maps of Indirect Standardized Drug Utilization Ratios of Raloxifene use among women ≥ 50 years by Primary Healthcare Zones in the region of Valencia, 2009. |
| --- |
| 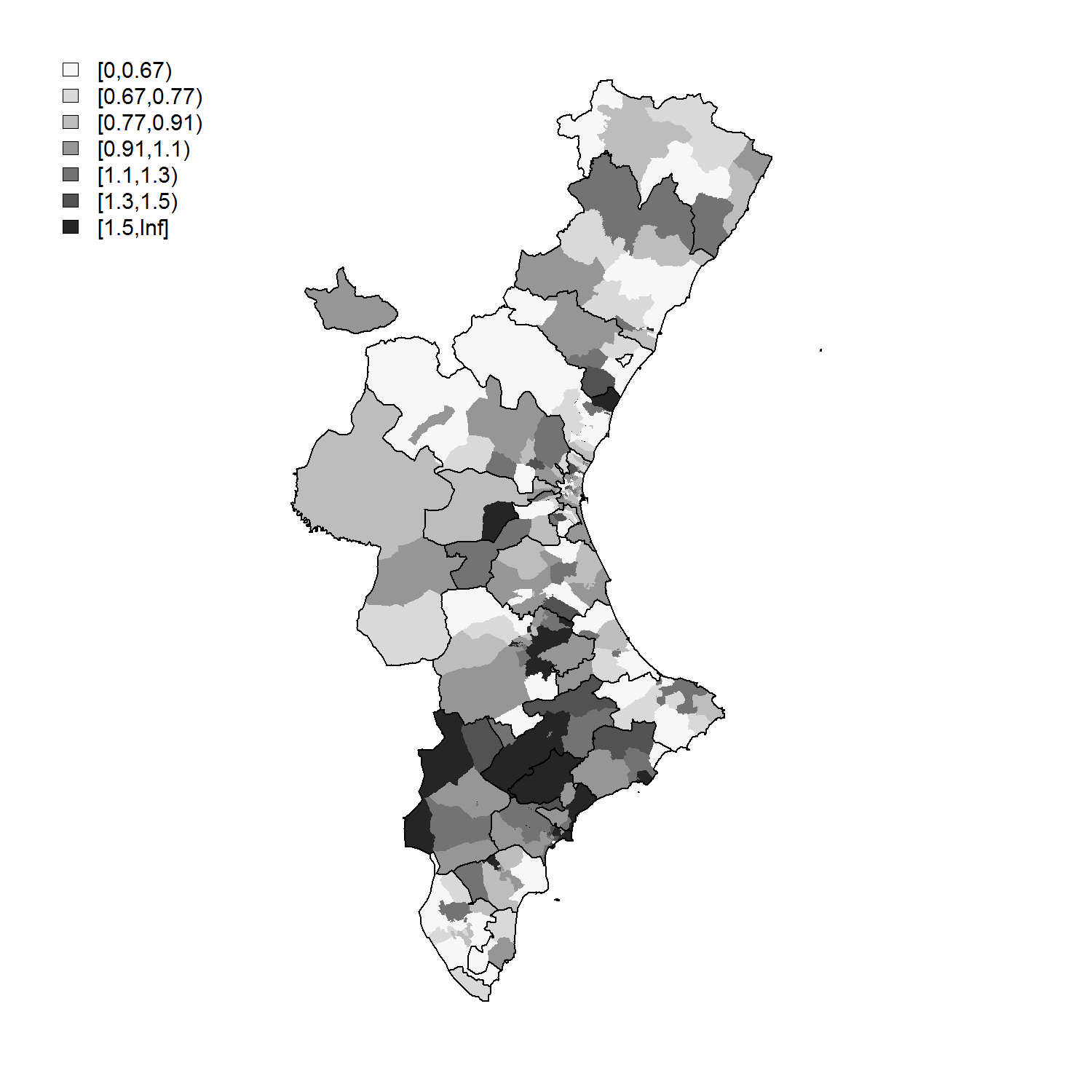 |
| *The lines on the map represent the geographical boundaries of the different Hospital Healthcare Departments (HHD). The cut-off points of Indirect Standardized Drug Utilization Ratios should be interpreted (like relative risks) on a ratios’ scale, corresponding to relative increases of consumption of 10% (1.10), 30% (1.30), 50% (1.50) and >50% (>1.50) with respect to the average of the Valencia region, and its symmetric negative -10% (0.91), -30% (0.77), -50% (0.67) and <-50 (<0.67). Map constructed upon raw maps from the Valencia Cartographic Institute (CC BY 4.0 © Institut Cartogràfic Valencià, Generalitat).* |

| Figure A2.4. Indirect Standardized Drug Utilization Ratios of Parathyroid Hormones use among women ≥ 50 years by Primary Healthcare Zones in the region of Valencia, 2009. |
| --- |
| 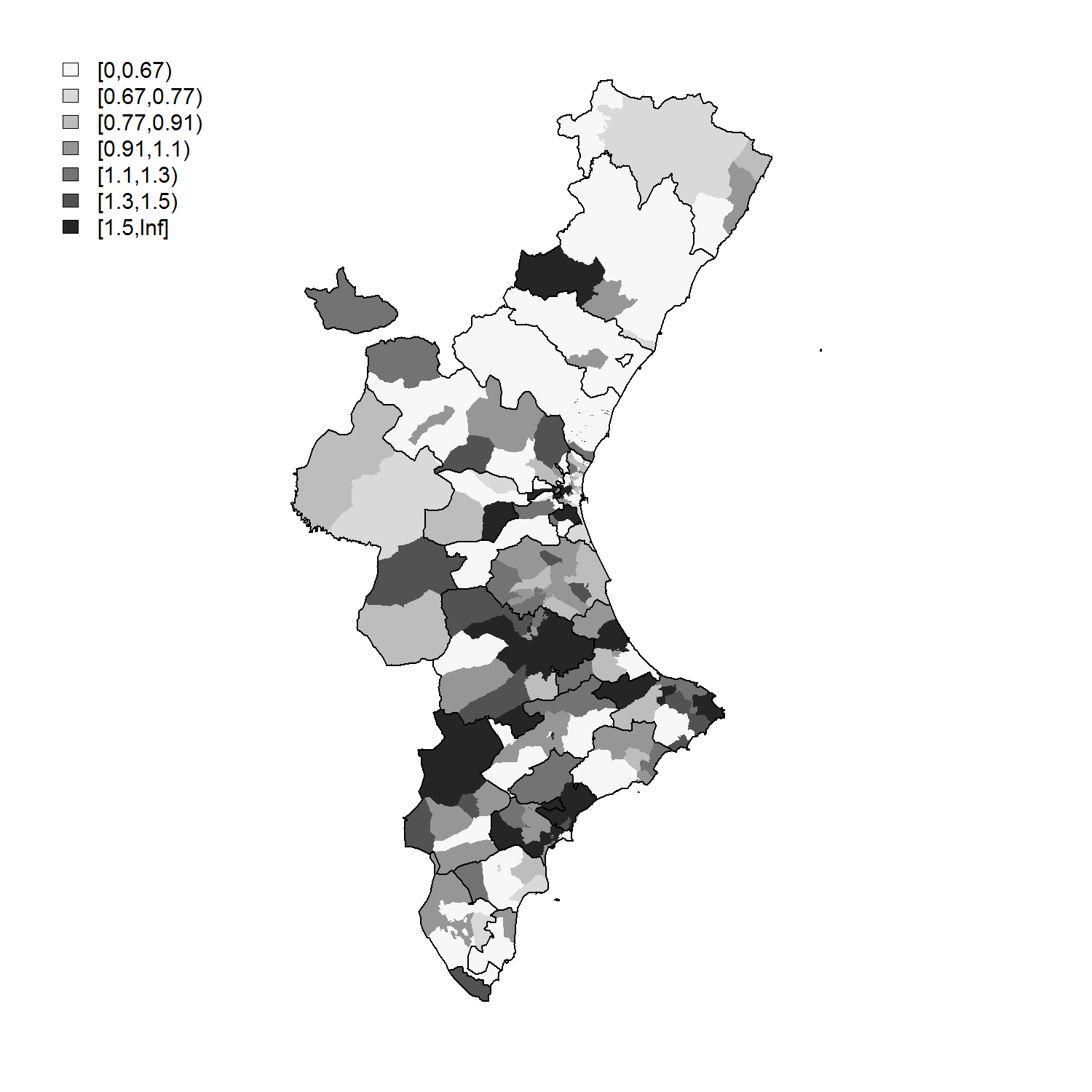 |
| *The lines on the map represent the geographical boundaries of the different Hospital Healthcare Departments (HHD). The cut-off points of Indirect Standardized Drug Utilization Ratios should be interpreted (like relative risks) on a ratios’ scale, corresponding to relative increases of consumption of 10% (1.10), 30% (1.30), 50% (1.50) and >50% (>1.50) with respect to the average of the Valencia region, and its symmetric negative -10% (0.91), -30% (0.77), -50% (0.67) and <-50 (<0.67). Map constructed upon raw maps from the Valencia Cartographic Institute (CC BY 4.0 © Institut Cartogràfic Valencià, Generalitat).* |

| Figure A2.5. Indirect Standardized Drug Utilization Rates of Calcitonins use among women ≥ 50 years by Primary Healthcare Zones in the region of Valencia, 2009. |
| --- |
| 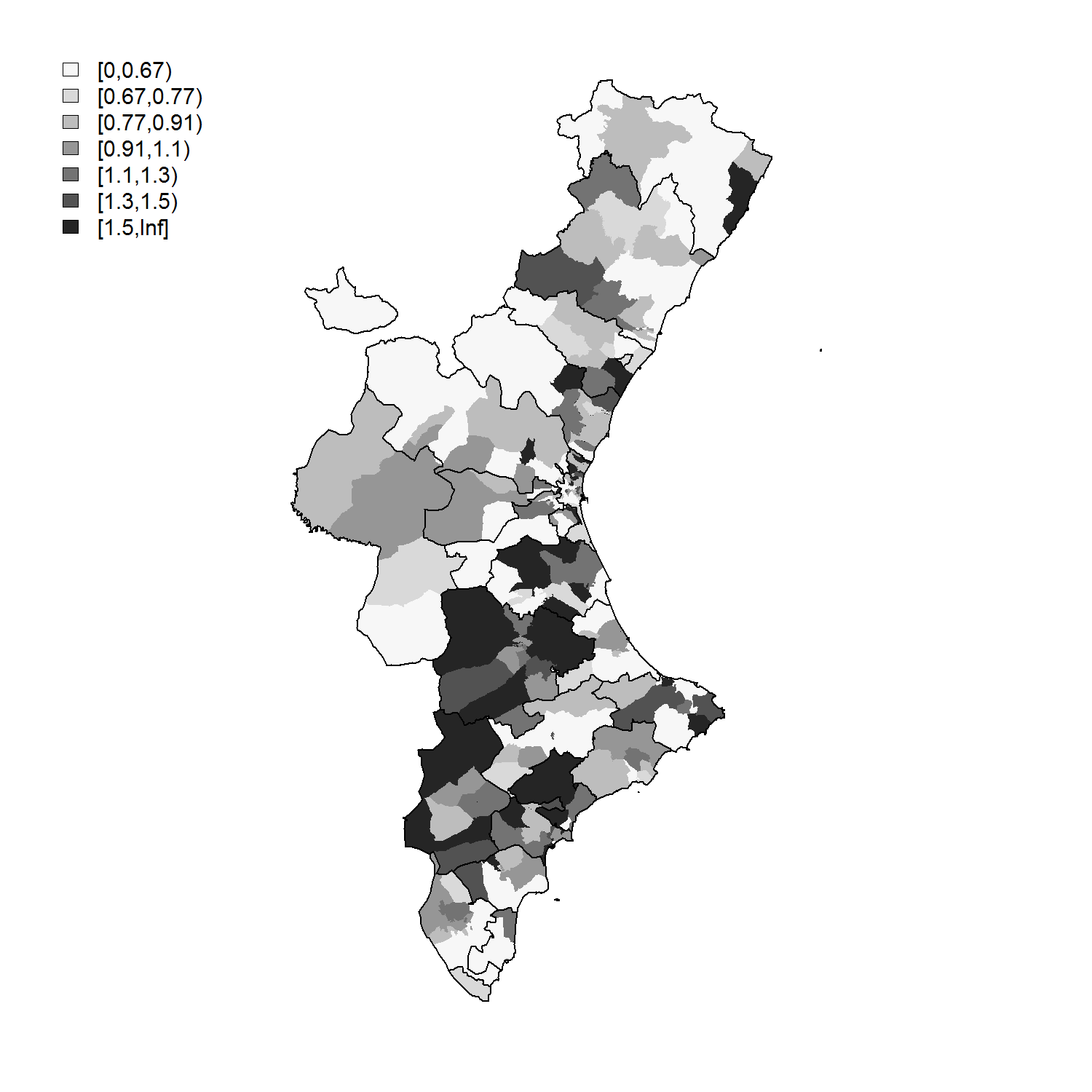 |
| *The lines on the map represent the geographical boundaries of the different Hospital Healthcare Departments (HHD). The cut-off points of Indirect Standardized Drug Utilization Ratios should be interpreted (like relative risks) on a ratios’ scale, corresponding to relative increases of consumption of 10% (1.10), 30% (1.30), 50% (1.50) and >50% (>1.50) with respect to the average of the Valencia region, and its symmetric negative -10% (0.91), -30% (0.77), -50% (0.67) and <-50 (<0.67). Map constructed upon raw maps from the Valencia Cartographic Institute (CC BY 4.0 © Institut Cartogràfic Valencià, Generalitat).* |

| Figure A2.6. Indirect Standardized Drug Utilization Rates of all osteoporosis medication use among women ≥ 50 years by Primary Healthcare Zones in the region of Valencia, 2009. |
| --- |
| 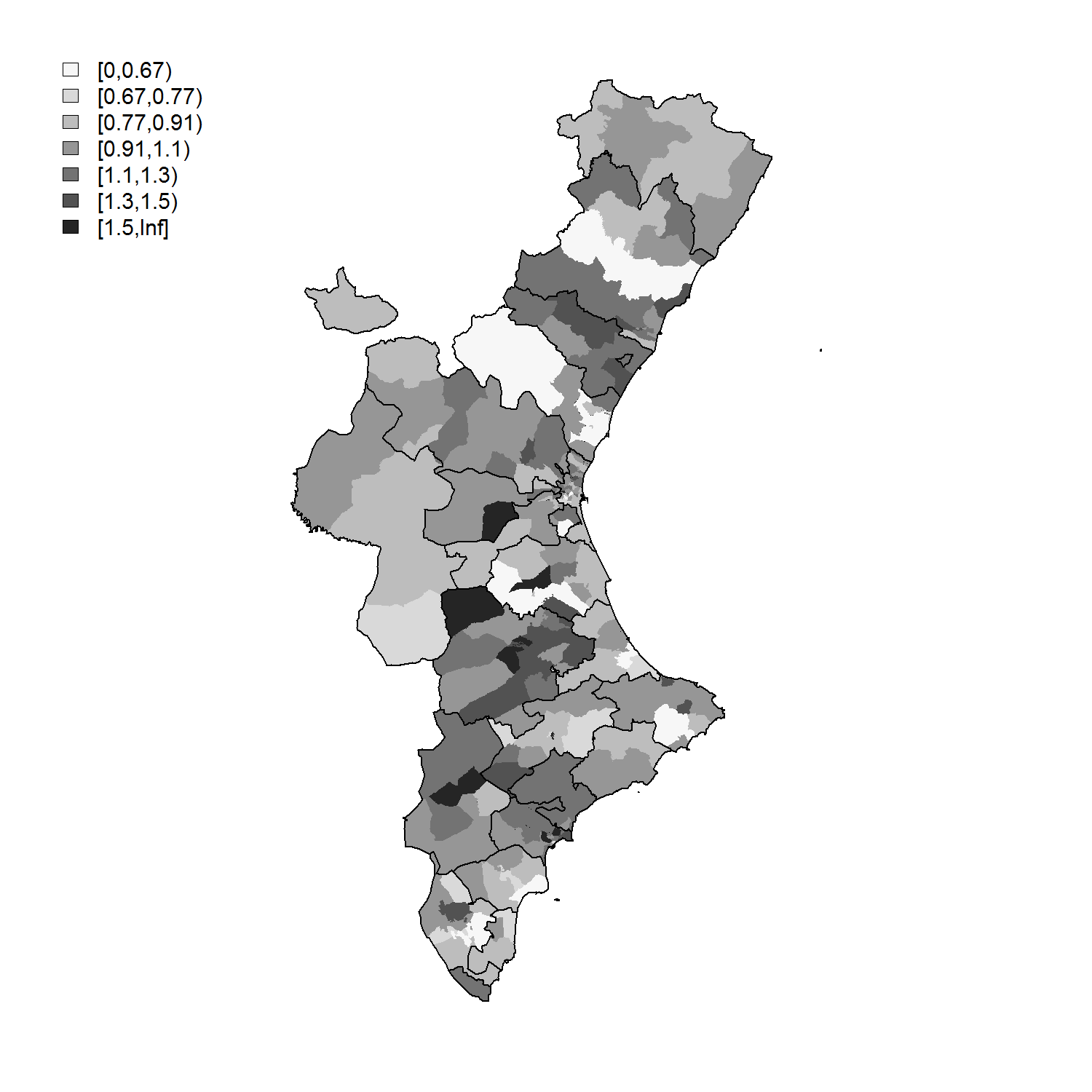 |
| *The lines on the map represent the geographical boundaries of the different Hospital Healthcare Departments (HHD). The cut-off points of Indirect Standardized Drug Utilization Ratios should be interpreted (like relative risks) on a ratios’ scale, corresponding to relative increases of consumption of 10% (1.10), 30% (1.30), 50% (1.50) and >50% (>1.50) with respect to the average of the Valencia region, and its symmetric negative -10% (0.91), -30% (0.77), -50% (0.67) and <-50 (<0.67). Map constructed upon raw maps from the Valencia Cartographic Institute (CC BY 4.0 © Institut Cartogràfic Valencià, Generalitat).* |
