# Supplementary material for: Variations in osteoporosis medication utilization. A population-based ecological cross-sectional study in the region of Valencia, Spain
Source: PLoS One. 2018 Jun 21;13(6):e0199086. doi: 10.1371/journal.pone.0199086 (PMC6013112; doi:10.1371/journal.pone.0199086)
Supplement: S3 Appendix — (DOCX) [file pone.0199086.s003.docx]

| Sanfélix-Gimeno G, Juliá-Sanchís ML, Librero-López J, Peiró S, García-Sempere A.  **Variations in osteoporosis medication utilization. A population-based ecological cross-sectional study in the region of Valencia, Spain** |
| --- |
|  |
| **S3 Appendix** |
| **Spearman correlations between Primary Healthcare Zones indirect standardized drug utilization ratios of the osteoporosis medication (Valencia region, 2009)** |
|  |

|  | Biphosph. | Strontium R | Raloxifene | Parathyr. H | Calciton. |
| --- | --- | --- | --- | --- | --- |
| Biphosphonates |  |  |  |  |  |
| Strontium Ranelate | **0.277**  *p<0.001* |  |  |  |  |
| Raloxifene | **0.098**  *p=0.129* | **0.151**  *p=0.019* |  |  |  |
| Parathyroid Hormones | **0.198**  *p=0.002* | **-0.016**  *p=0.799* | **0.198**  *p=0.002* |  |  |
| Calcitonins | **0.270**  *p<0.001* | **0.140**  *p=0.030* | **0.241**  *p<0.001* | **0.349**  *p<0.001* |  |
|  | | | | | |
