# Supplementary material for: Variations in osteoporosis medication utilization. A population-based ecological cross-sectional study in the region of Valencia, Spain
Source: PLoS One. 2018 Jun 21;13(6):e0199086. doi: 10.1371/journal.pone.0199086 (PMC6013112; doi:10.1371/journal.pone.0199086)
Supplement: S4 Appendix — (DOC) [file pone.0199086.s004.doc]

| Sanfélix-Gimeno G, Juliá-Sanchís ML, Librero-López J, Peiró S, García-Sempere A.  **Variations in osteoporosis medication utilization. A population-based ecological cross-sectional study in the region of Valencia, Spain** | |
| --- | --- |
|  | |
| **S4 Appendix** | |
| **Median Odds Ratio of osteoporosis medication use (DDD/1000/Day) among women ≥50 years by Hospital Departments in the Valencia region, 2009.** | |
| Biphosphonates | Strontium ranelate |
| 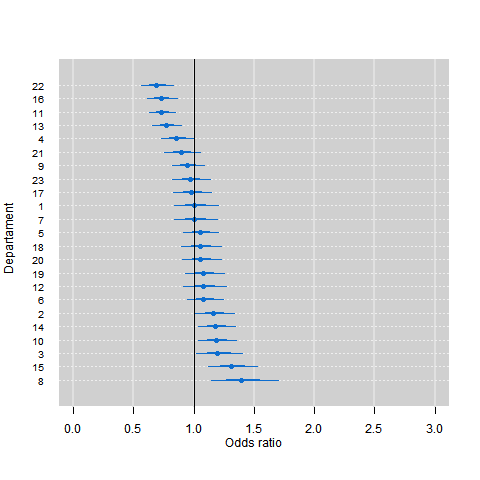 | 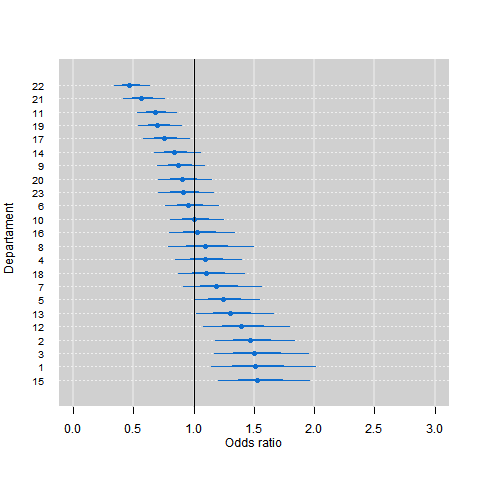 |
| Raloxifene | Parathyroid Hormones |
| 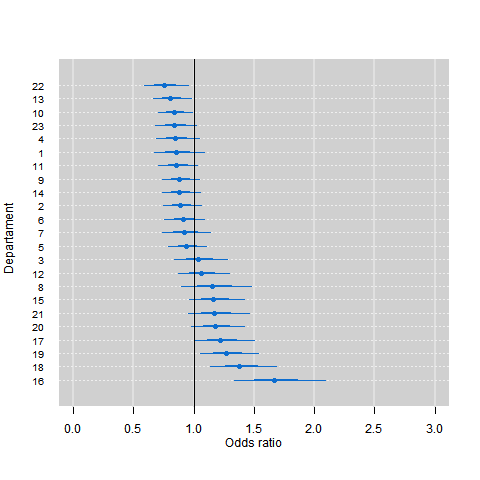 | 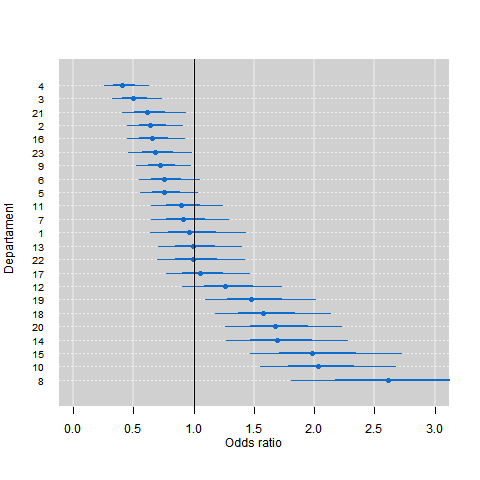 |
| Calcitonins | All anti-osteoporotic drugs |
| 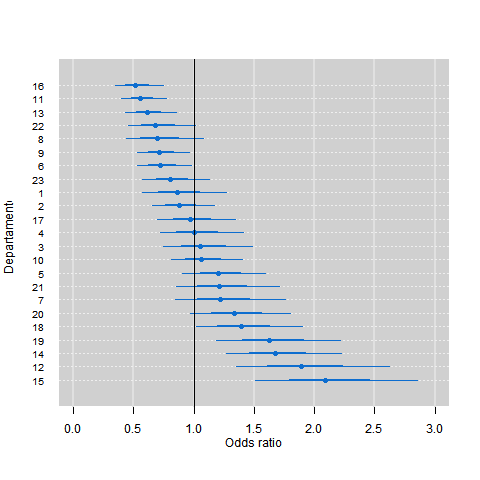 | 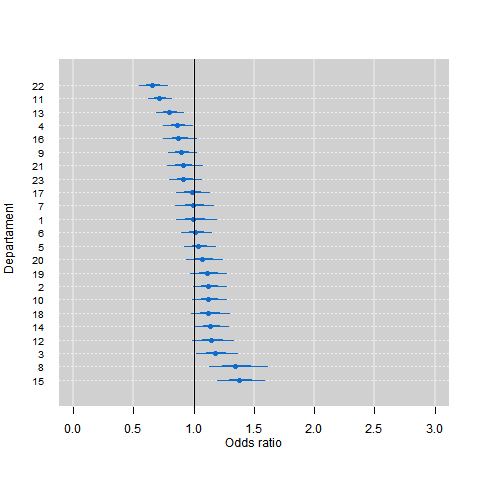 |
| *Median Odds Ratio derived from Oneway ANOVA random effects (see methods section). Reference represents the regional average.* | |
|  | |
